# The structure of the bacterial outer membrane transporter FusA enabled by addition of the native lipid lipopolysaccharide

**DOI:** 10.1016/j.yjsbx.2025.100141

**Published:** 2025-11-18

**Authors:** Jonathan M. Machin, Khedidja Mosbahi, Dheeraj Prakaash, Sheena E. Radford, Daniel Walker, Antreas C. Kalli, Neil A. Ranson

**Affiliations:** a Astbury Centre for Structural Molecular Biology, School of Molecular and Cellular Biology, Faculty of Biological Sciences, University of Leeds, LS2 9JT, United Kingdom; b School of Infection and Immunity, College of Medical, Veterinary, and Life Sciences, University of Glasgow, G12 8QQ, United Kingdom; c Astbury Centre for Structural Molecular Biology and Leeds Institute of Cardiovascular and Metabolic Medicine, School of Medicine, University of Leeds, LS2 9JT, United Kingdom; d Strathclyde Institute of Pharmacy and Biomedical Sciences, University of Strathclyde, G4 0RE, United Kingdom

**Keywords:** Structural biology, cryoEM, Membrane protein, Bacterial membrane protein, Transporter, Ferredoxin, Ton-b dependent transporter, Lipopolysaccharide

## Abstract

Lipopolysaccharide (LPS) is a glycolipid found uniquely in the outer membrane of diderm bacteria, formed of 4–7 acyl chains covalently linked to an extended polysaccharide chain. While a few examples of the interaction between LPS and outer membrane proteins (OMPs) have been structurally characterised, either experimentally or computationally, the precise nature of LPS-OMP interactions and their functional consequences remains unclear. Here, we show that the addition of LPS facilitated cryoEM structure determination of FusA, a 100 kDa TonB-dependent outer membrane transporter from *P. atrosepticum*. A 2.8 Å structure combined with molecular dynamics of FusA with different LPS models reveals LPS binding sites with a strong LPS interaction site located adjacent to the β-seam region of the FusA β-barrel. The requirement of lipid binding for successful structure determination indicates a stabilisation of the protein, which in turn suggests a potential method for solving other, small OMPs and membrane proteins. Further, it hints at how LPS may mediate protein conformation and thus how LPS and OMPs can work in concert to maintain a structural and functional OM.

## Highlights

• The structure of FusA, a 100 kDa bacterial outer membrane protein, was intractable by cryoEM.
• Potential lipopolysaccharide binding sites were identified by course-grained MD simulation.
• Doping of purified LPS into detergent-solubilised FusA facilitated a 2.8 Å resolution structure by cryoEM.
• The structure included bound LPS molecules.

## Graphical abstract

**Figure:**
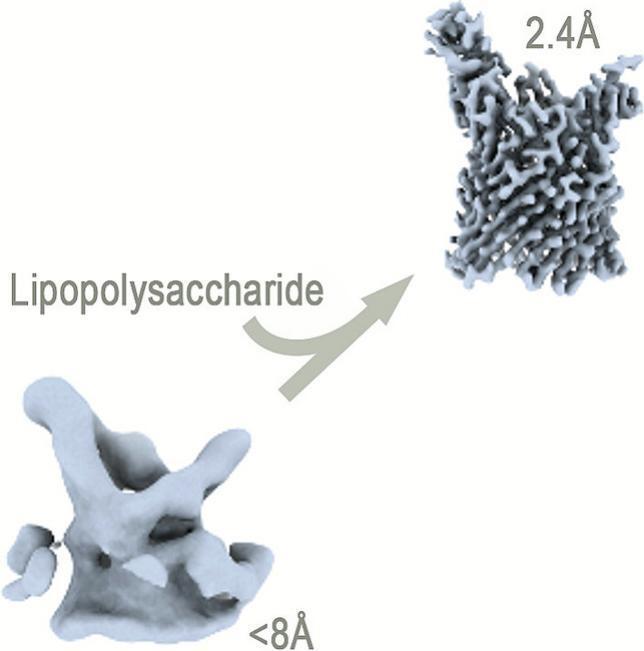

## Introduction

Outer membrane proteins (OMPs), the transmembrane proteins found in the outer membrane (OM) of diderm bacteria, represent an attractive drug target owing to their cell-surface accessibility and the range of essential and virulence-related functions that they perform (Delcour, 2009, Ellis and Kuehn, 2010, Xu et al., 2020). Nearly all OMPs adopt a β-barrel architecture, in which transmembrane β-strands are linked by shorter intracellular turns and longer extracellular loops (Fairman et al., 2011). OMPs sit within the OM, which consists of a unique, highly asymmetric membrane, with phospholipids dominating the inner (periplasmic facing) leaflet and lipopolysaccharide (LPS) exclusively in the outer leaflet (Horne et al., 2020). LPS is composed of 4–7 variably-long acyl chains, and an extracellular sugar chain consisting of a well-conserved 6–7 sugars close to the cell (the core sugars) and a highly diverse repeating unit extending away from the membrane (the O-antigen) (Basauri, 2020, Liu, 2020). Membrane asymmetry is strictly controlled (Abellon-Ruiz, 2023), and LPS is known to have a unique binding fingerprint to a range of OMPs (Shearer et al., 2019). LPS has also been shown to be important for the oligomerisation (Arunmanee, 2016) and function of specific OMPs (Nyenhuis et al., 2020, Patel, 2016). Despite these findings, much remains unclear about the nature of LPS-OMP interactions, the sequence/structural determinants and motifs for LPS binding by proteins, or how LPS may interact with antibiotics or mediate resistance (Manioglu, 2022).

Similar to other membrane proteins (Nygaard et al., 2020); cryoEM has transformed the study of OMPs, for example revealing important details about OMP insertion into the membrane via the β-barrel assembly machinery (Doyle, 2022, Shen, 2023, Fenn, 2024) and the mechanisms of action of oligomeric transporters (White, 2023). Despite these successes, the majority of OMPs remain challenging targets for cryoEM owing to their small size, β-strand architecture and the rotational pseudo-symmetry of their barrel domains which makes particle alignment during cryoEM refinements challenging. Recently solved structures of smaller, monomeric OMPs have been achieved by exploiting native or synthetic protein binding partners, including macrobodies (Botte, 2022), lipoproteins (Iadanza, 2016) and phage components (van den Berg et al., 2022). Many OMPs that have had their structures solved by cryoEM are TonB-dependent Transporters (TBDTs), including both dimeric (White, 2023, Madej, 2020) and monomeric (Abellon-Ruiz, et al., 2022) examples. TBDTs are a broad class of nutrient active transporters that couple the proton motive force across the inner membrane with active substrate transport across the OM, via the periplasm spanning TonB protein and inner membrane ExbBD motor complex (Noinaj et al., 2010). Importantly, the TBDTs BtuB and FhuA are known to make structurally and functionally-important interactions with LPS, and both have been experimentally demonstrated either indirectly with EPR (BtuB (Nyenhuis et al., 2020, Nyenhuis, 2020)) or structurally resolved by crystallography and EM (FhuA (van den Berg et al., 2022, Ferguson, 2001)).

A recently discovered TBDT family scavenges iron-containing proteins from the host and imports them to the periplasm, where they are proteolytically degraded, and their iron released for use by the bacterium (Grinter, 2019). These protease-associated import systems are wide-spread in diderm bacteria and include the *E. coli* transporter/protease system YddB/PqqL, which is important for uropathogenic fitness (Subashchandrabose et al., 2013, Wurpel et al., 2015), and has been linked to the import of the antibiotic novobiocin (Choi and Lee, 2019). The best-studied member of this class of TBDT is FusA from plant-pathogenic *Pectobacterium* spp., which functions in concert with the TonB-like protein FusB and the protease FusC to import and cleave plant ferredoxin (10 kDa), thereby releasing iron from this abundant, iron–sulphur cluster-containing host protein (Wojnowska and Walker, 2020, Mosbahi et al., 2018). A previously reported crystal structure showed that the extracellular loops of FusA are unusually extensive, at least in part to help form a binding site for the large ferredoxin substrate (Grinter, 2016). These loops increase the size of FusA compared to many other OMPs, making it more amenable to cryoEM, as well as providing a large surface for possible LPS interactions. Interestingly, the ferredoxin uptake system (FUS) can be exploited by antibacterial proteins such as the bacteriocins pectocin M1 and M2. These unusual bacteriocins contain an N-terminal ferredoxin domain with high levels of sequence and structural homology to plant ferredoxin enabling them to parasitise the FUS and penetrate barrier of the OM via FusA (Grinter et al., 2012).

Here we demonstrate that although extensive initial efforts to solve the structure of apo-FusA by cryoEM were unsuccessful, guided by molecular dynamics (MD) simulations that indicated strong LPS binding sites on the FusA structure, addition of LPS to detergent-solublised FusA allowed a 2.8 Å structure of FusA to be determined. The observed conformation of FusA includes resolved sugars from the bound LPS moieties which both validate and finesse the LPS-OMP interactions suggested by MD. Together, these results suggest the potential for LPS to modulate OMP structure, and highlight the need to consider the role of this lipid when studying OMP structure and function.

## Results

### The structure of apo-FusA could not be solved by cryoEM

TBDTs expressed as inclusion bodies can be readily refolded to their native conformation in detergent micelles, an approach that has facilitated the structures of many TBDTs to be solved via macromolecular crystallography, including FusA (Grinter, 2016). Prior to cryoEM imaging, FusA was refolded into LDAO micelles (LDAO micelles are typically beneficial for solving the structure of smaller membrane proteins by cryoEM owing to their relatively small size compared to many other detergent micelles). The refolded apo-FusA was then imaged using cryoEM, but high-resolution structure determination was not successful, and only an ∼ 13Å structure was resolvable.

**Fig. 1:**
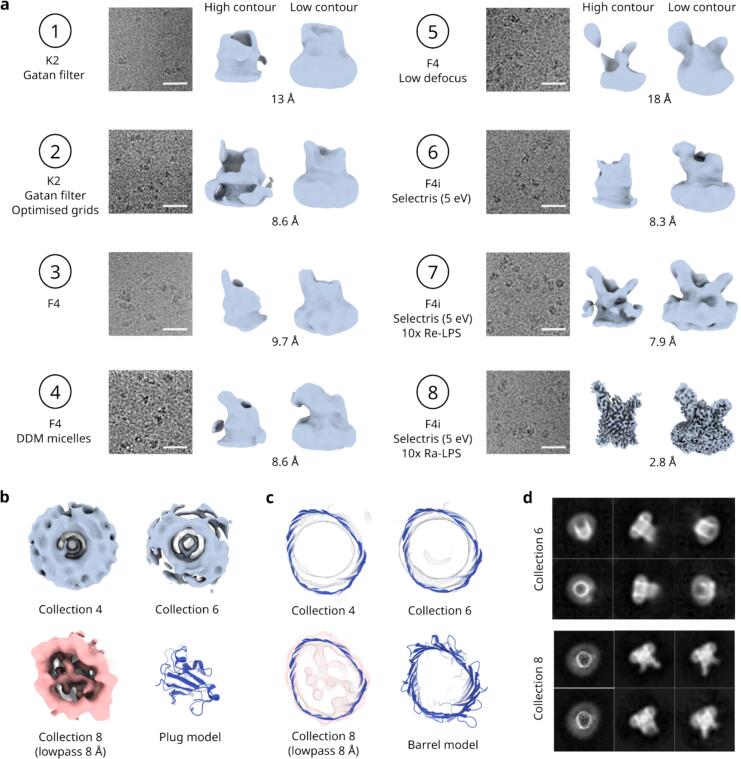
FusA structure determination is facilitated by addition of LPS. (a) Apo-FusA across multiple collections (numerical indicators used throughout text and methods) with various hardware and data collection parameters did not resolve (1–6, nominal resolutions reported). Upon addition of Re-LPS (7) or Ra-LPS (8) FusA resolved to low-resolution and high resolution respectively. High-contour (left) and low-contour (right) representations of each model are presented. Scale bars are 25 nm. In collections 1–6, rotational averaging was observed in (b) the plug domains and (c) the barrel of apo-FusA models with the formation of symmetrical cylinder-like density (upper) rather than the expected asymmetric shape resolved upon the addition of LPS (lower). (d) Comparison of best 2D classes from apo-FusA (upper) and Fusa:Ra-LPS (lower). Apo-FusA classes highlight the alignment to the pseudo-symmetry axis by the lack of detail and presentation of symmetrical features (compared to expected). All boxsizes are18.2 nm.

To overcome these initial problems, we attempted to improve image contrast by optimising grid preparation to ensure evenly thin ice (final conditions including an amylamine glow-discharge and a 10 s pause between sample application and blotting). We also used more modern EM detectors (Falcon 4i with and without Selectris energy filter). Both changes improved the signal to noise ratio of cryoEM data and should in principle have made the particles of FusA more alignable during the cryoEM refinement. However, while these optimisations yielded marginally improved 2D classes, none resulted in substantially better reconstructions (Fig. 1a, collections 1–6).

Upon inspection of the 3D reconstructions generated from these data, it was observed that the plug domain that sits within the centre of the barrel was forming cylindrical density, rather than adopting its expected asymmetric shape (Fig. 1b). In addition, the transmembrane barrel had a circular cross-section, rather than the expected irregular elliptical shape described by the X-ray crystal structure (Fig. 1c). Together, these observations suggested that the images were being inappropriately rotationally averaged, with the low-resolution pseudo-rotational symmetry axis down the centre of the barrel overwhelming the rest of the protein signal, thus preventing alignment on higher-resolution, asymmetric features. This is further supported by inspection of the 2D classes (classified from particles contributing to the best final model), which show strong signal for the barrel (which was circular in top-views), but only weak, featureless density for the plug and extracellular loop regions of the protein (Fig. 1d, upper), compared to the asymmetric and strong signal expected (Fig. 1d, lower). Adjustment of the imaging parameters, including low-defocus and stage tilt failed to break the inappropriate rotational-averaging applied to the models. Solubilising FusA in DDM detergent rather than LDAO also did not help. Thus, despite extensive efforts (see Fig. 1 in its totality and Table S1), and a deep experience in solving the structures of other β-barrel OMP structures (e.g. (Fenn, 2024, White, 2023, Iadanza, 2016, Madej, 2020, White, 2021), solving the structure of apo-FusA by cryoEM remained intractable.

### Molecular dynamics simulations identified specific LPS binding sites

**Fig. 2:**
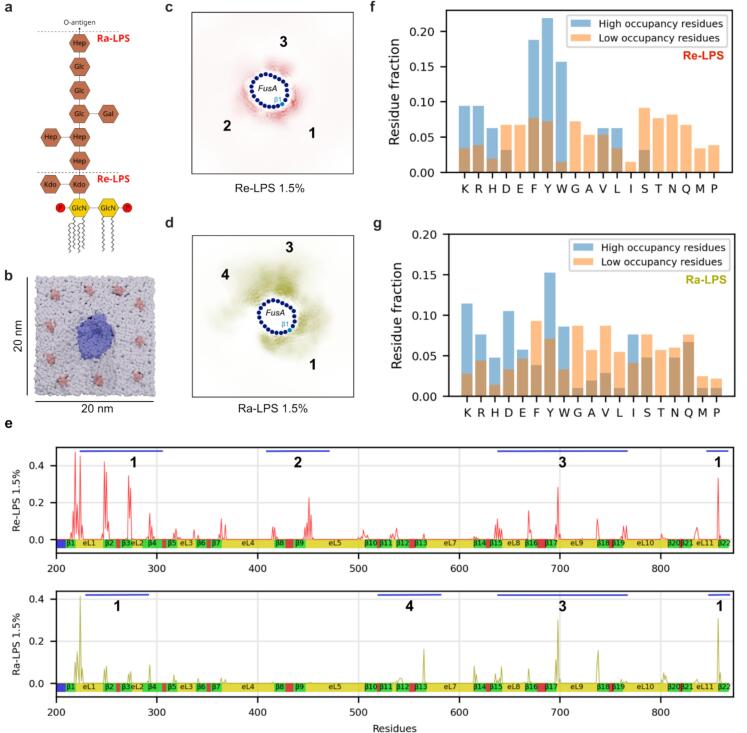
CG-MD simulations reveal specific Re-LPS and Ra-LPS binding to FusA. (a) Structure of *E. coli* Re-/Ra-LPS. (b) The starting frame for the sparse LPS (∼1.5 %) simulations (LPS: pink, FusA: blue, phospholipid: gray). (c) Re-LPS and (d) Ra-LPS lipid density plots averaged over the second half of the simulation time. Frames were aligned on the FusA protein using the protein backbone particles for the alignment. Approximate location of β-strands indicated by blue dots, progressing in a clockwise direction from β1 to β22. Distinct binding sites are numbered 1–4 as in (e). (e) Normalised (by lipid count and number of frames) lipid-protein contacts between FusA and Re-LPS (upper, red) and Ra-LPS (lower, green). Binding sites numbered as in (c)/ (d). FusA residue identity interacting with (f) Re-LPS or (g) Ra-LPS in either high (>25 % simulation time) or low (<5% simulation time) occupancy binding as indicated by pyLipID. Residue fraction corresponds to the fraction of each residue group. (Number of residues per group: Re-LPS: low = 32, high = 209; Ra-LPS: low = 105, high = 368). (For interpretation of the references to colour in this figure legend, the reader is referred to the web version of this article.)

Some OMPs, including the TBDTs BtuB and FhuA, are known to interact specifically with LPS in the OM (Nyenhuis, 2020, Ferguson, 2001) (K12 *E. coli* LPS shown in Fig. 2a). To probe how LPS might interact with FusA, coarse-grained molecular dynamics (CG-MD) simulations were performed. LPS with polysaccharides of different lengths have been previously parameterised, including two truncated versions: Re-LPS (with no sugar extensions) and Ra-LPS (with all the core-sugars but no O-antigen) (Hsu et al., 2016). Lipid only simulations with 1:3 Re/Ra-LPS:DMPE in the outer leaflet and 80:15:5 DMPE:DMPG:cardiolipin in the inner leaflet (matching native inner leaflet headgroup composition (Horne et al., 2020) showed the expected slow diffusion and clustering of the LPS moieties in the membrane (Fig. S1), with Ra-LPS being more prone to both these features than Re-LPS. To overcome the challenges of the slow diffusion time of LPS (see Methods), FusA was simulated with 10 Re-LPS or Ra-LPS LPS moieties (∼1.5 % molar concentration) initialised singularly in a distant ring around the protein (Fig. 2b). Systems were found to have converged after 10 µs for Re-LPS and 20 µs for Ra-LPS (Fig. S2; S3). In all simulations, similar to previously reported data, cardiolipin in the inner leaflet is excluded from positions under LPS molecules in the outer leaflet. (Shearer et al., 2019).

Assessing the average LPS density around the protein over the simulation time demonstrates a strong preference for LPS to interact with FusA, while the bulk lipid area has negligible Re-LPS density. Different patterns of preferential binding around FusA are observed for each LPS type (Fig. 2, Fig. 2). To elucidate these sites, the contacts between the phosphates of the LPS and each protein residue were determined (Fig. 2e, upper). Re-LPS interactions are dominated by three specific regions around the protein: site 1 at the β-seam (strands β1-3, β22), site 2 at strands β8-9 and site 3 at strands β15-18. All of these interactions are adjacent to positively charged residues in FusA, with the site at the β-seam having the highest number of interactions (Fig. S3). Ra-LPS, with a full sugar-core that extends several nm away from the hydrophobic core of the membrane, is able to form larger surface contacts with FusA. The protein-lipid interaction analyses demonstrates that while sites 1 and 3 (as identified for Re-LPS:FusA) are maintained by Ra-LPS, site 2 is missing and a new interaction location is observed, site 4 (Fig. 2e, lower**).** These data, coupled with the differing ratios between the number of interactions at each site, further suggest that there are LPS type-specific interactions with FusA, presumably mediated by the extended core polysaccharide chain present in Ra-LPS.

To study further the interaction between FusA and LPS, LPS binding poses were assessed using pyLipID (Song, 2022), identifying six poses for Re-LPS and 8 for Ra-LPS, around FusA’s barrel (Fig. S4). Comparing the residues that interact with a high percentage occupancy over the simulation (>25 %) and those with a low occupancy (<5%) highlights the residues important for mediating LPS binding. For Re-LPS high occupancy binding is dominated by positively charged and aromatic residues (Fig. 2f), which is not unexpected owing to the negatively charged phosphates on the LPS and the enrichment of aromatic amino acid side chains around the hydrophobic membrane interface in OMPs (Jackups and Liang, 2005). Ra-LPS also shows enrichments of positive and aromatic residues but some differences are observed from Re-LPS (Fig. 2g). Given that Ra-LPS contains the Re-LPS moiety, the differences inform on the binding of the extended polysaccharide of Ra-LPS, in particular its preference for isoleucine, aspartic acid and glutamic acid, which are enriched in both high occupancy binding sites and in Ra-LPS compared to Re-LPS.

#### Addition of LPS to detergent-solublised FusA facilitates high-resolution structural solution

**Fig. 3:**
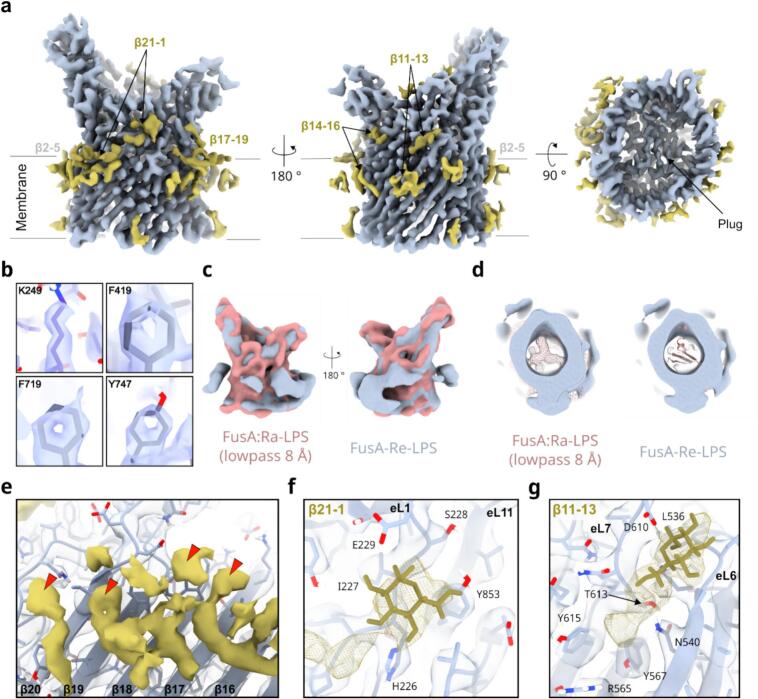
High resolution FusA:Ra-LPS structure shows LPS binding. (a) Overview of cryoEM map showing protein density (blue) and non-protein density (green). Assigned LPS binding sites are labelled as in main text. (b) Details of well-resolved side chains showing quality of the cryoEM density. (c) Comparison of overall shape and structure of FusA:Re-LPS (blue) and FusA:Ra-LPS (pink) lowpass filtered to an equivalent density shows similar barrel shapes, while (d) the plug domain in FusA:Re-LPS appears to be missing compared to lowpass filtered FusA:Ra-LPS (left) or the modelled plug (right). (e) Details of acyl-chains assigned to LPS, ring-shaped densities at the top of the acyl chains likely corresponding to sugars are marked with a red triangle. Details of extra-micellar non-protein density at the (f) β21-β1 and (g) β11-13 LPS sites. Glucose molecules are modelled for size comparison only. See also Fig. S9. (For interpretation of the references to colour in this figure legend, the reader is referred to the web version of this article.)

Given the specific and apparently strong LPS-FusA interactions indicated by the CG-MD simulations, Ra-LPS and Re-LPS from K12 *E. coli* were doped into the LDAO micelles of FusA at a 10:1 LPS:protein ratio. The structure of LPS is well conserved between *E. coli* (Bertani and Ruiz, 2018) and *Pectobacterium carotovorum* (Fukuoka, 1997) with the Lipid-A moiety, and the first four sugars of the polysaccharide chain being identical, with variation in the further-extended and branching portion of the molecule (Fig. S5), and thus interactions are expected to be similar. CryoEM micrographs of the LPS-FusA data were not superficially different from previously collected datasets (Fig. 1a), and initial 2D classification yielded similar looking classes to those previously obtained. However, following multiple rounds of divisive 2D classification on specific views of the protein, high-resolution, asymmetric classes were obtained (Fig. 1d). Training particle picking models explicitly on the different views represented by these classes generated sub-datasets, that when recombined, yielded enough particles to reconstruct models for both the Re-LPS and Ra-LPS datasets. The Ra-LPS dataset, for which about twice as many images were obtained (∼8 000 micrographs vs ∼ 4000 micrographs), finally resolved to a global resolution of 2.8 Å (Fig. 3a) with the core of the structure extending to ∼ 2.2 Å (Fig. 3b), and the Re-LPS data to ∼ 7.9 Å (Fig. S6**, 7)**. The structure of the protein, especially in the plug and transmembrane barrel region, is very similar to the previously solved crystal structure (average RMSD: 1.2 Å, Fig. S8). The barrel shape of FusA:Re-LPS is consistent with FusA:Ra-LPS filtered to an equivalent resolution (Fig. 3c). Surprisingly, there is a greatly reduced amount of density for the plug (Fig. 3d), suggesting that it has been at least partially dislodged from the pore, although it is uncertain if this is biologically relevant. Altering the number of particles in the FusA:Ra-LPS reconstruction to match the other collections did not preclude a high-resolution solution (Fig. S6**, 7**), and refining the optimised particles from collections 1–6 against the high-resolution model failed to yield a better solution. Together, this indicates both that the presence of the LPS is likely critical to enable structure determination, and that the nature of the FusA-LPS interactions may alter the location of the plug in the FusA barrel.

Consistent with these conclusions, non-protein density was observed at a variety of sites around the reconstruction, both in the micelle and interacting with the protein surface (Fig. 3a**, S9a**). Accurate identification of non-protein density was possible owing to the high quality of the protein portions of the map in these regions, which allowed confident side-chain placement for nearly all residues in the regions of interest. LPS was conservatively assigned if density could be observed for both its acyl chains and adjacent extra-micellar density that could belong to the polysaccharide chain. In some cases, ring shaped moieties capped the top of acyl-chain density in the micelle (e.g. Fig. 3, Fig. 3.f. **S9c**): these features must arise from the LPS sugar rings given that the solubilising detergent (LDAO) contains no ringed chemical groups. While extra density, attributable to LPS sugars, was observed binding to the extracellular loops of the protein (e.g. Fig. 3, Fig. 3, **S9e**, glucose sugars in density shown for reference only), the regions of density were disconnected, indicative of stronger binding sites for specific LPS sugars on the protein surface anchoring a more weakly associated polysaccharide in place. This made determining the path of the polysaccharide chain impossible, and thus LPS was not explicitly modelled. However, the assigned LPS density did appear to cluster into four specific regions (Fig. 3a**, S9a**) which were labelled according to the likely interacting β-strands at the membrane interface (β11-13, β14-16, β17-19, β21-β1; see Fig. 3**e-g**, **S9**). In addition, a cluster of acyl chains, without corresponding extra-micellar density, was observed at β2-5 (Fig. S9**c**). The experimental conditions used mean that doped-in Ra-LPS could freely associate with the extracellular or intracellular side of the barrel, but there are many fewer well-resolved acyl-chains on the intracellular side. While the acyl-chains alone cannot be assigned between LPS and LDAO, this does suggest a preference for the LPS bind to its native, extracellular position.

Closer inspection of the protein side chains that interact with the extra-density reveals binding sites that are enriched in positive charges, and the identity of many of the interacting residues is consistent with those identified by MD to be enriched in LPS binding sites (e.g., Fig. 3, Fig. 3**, S9e** c.f. Fig. 2, Fig. 2). In several cases, clusters of aromatic residues appear to bind the sugar moieties within Lipid-A and/or its adjacent acyl-chain regions. While the presence of aromatic residues is insufficient for LPS binding (FusA has a strong aromatic girdle, with many aromatic residues not apparently interacting with LPS), they do appear able to form important stabilising interactions (e.g., Fig. 3e**, S9b**), supporting the enrichment observed by MD. In addition, grooves on both the transmembrane and extracellular portions of the protein help hold the LPS in position, demonstrating the importance of both steric and chemical considerations for LPS binding (Fig. S9**f**). Indeed, much of the resolved density sits within binding pockets in the protein, further supporting a model of a several strong binding sites linked by only weakly-associated polysaccharide chain. Despite the addition of LPS being required for successful structure determination, no major structural modulation is observed in FusA around the LPS binding sites in comparison to the apo crystal structure (Fig. S8). There are small differences in the extracellular loops between the two structures, and suggestively the tip of eL8, which sits directly above the LPS bound at β11-13, has rotated ∼ 1 nm outwards compared to the crystal structure, a movement that may plausibly be induced by interactions with the LPS polysaccharide.

#### EM and MD simulations reveal consistent patterns of LPS binding

**Fig. 4:**
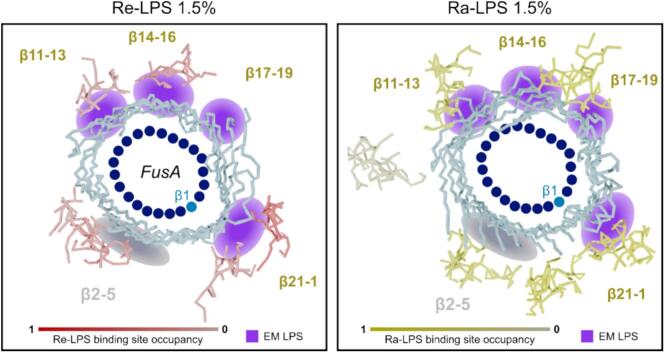
Comparison of Ra-LPS binding to FusA by CG-MD and cryoEM. Consensus LPS-FusA binding poses determined by pyLipID for the sparse Re-LPS (pink-red, left) and Ra-LPS (yellow-green, right) simulations coloured by the normalised binding site occupancy. FusA transmembrane strands are shown in blue and the location of β-strands indicated by blue dots, progressing in a clockwise direction from β1 to β22. The approximate locations of the LPS binding sites inferred from the cryoEM are indicated by purple spots and labelled as in the main-text. The grey spot at β2-5 is where acyl-chains were observed binding without additional non-micellar density. (For interpretation of the references to colour in this figure legend, the reader is referred to the web version of this article.)

Owing to the incomplete resolution of the LPS by cryoEM, it is only possible to broadly compare it to the LPS binding observed in the CG-MD simulations. Taking the consensus FusA:LPS binding poses determined by pyLipID from each simulation set and comparing them to the approximate location of the LPS indicated by the EM (Fig. 4) shows that there is overlap in the determined LPS binding sites. Comparing the FusA residues identified in by our LPS-protein contacts to form high number of interactions with LPS shows similar matching patterns (Fig. S10**)**. Both the Re-LPS and the Ra-LPS simulations match at three of the four sites, with the Re-LPS showing minimal additional binding, and the Ra-LPS interacting at least one other location (Fig. 4). Both the Ra-LPS and Re-LPS simulations show LPS binding at the β2-5 site, for which only acyl-chain density was observed by EM, suggesting that this density is likely a *bona fide* LPS. It is intriguing that while the high-resolution structure was obtained with Ra-LPS, the Re-LPS simulations identified the binding sites marginally better, suggesting that the extra sugars may not alter the binding mode to FusA substantially. Indeed, the strong correlation between binding sites in the Re-LPS simulation and the cryoEM density indicate that binding is being driven by Lipid A, rather than by the extended polysaccharide chain (supported by the relative paucity of extramicellar density observed by cryoEM). Differences between the simulations and the EM (and between the Re-LPS and Ra-LPS simulations) may emerge due to differing affinities at the binding sites to the two types of LPS, as well as different interactions with LDAO and lipid in the EM and simulations respectively. Also, the Ra-LPS polysaccharide, which tended to collapse when the LPS molecules are isolated (the average length of the polysaccharide at the start of simulation is 2.6 nm vs 1.5 nm at the end of the simulation), may plausibly preclude some native interactions with the protein. Despite these possible limitations, the CG-MD approach successfully identified the majority of the LPS binding sites, as verified by the experimentally determined structure.

## Discussion

Despite our initial inability to solve the structure of apo-FusA using cryoEM, guided by molecular dynamics simulations that indicated strong and specific LPS binding to FusA, the addition of LPS to detergent-solublised FusA facilitated high resolution structural solution and visualised LPS binding. The addition of LPS likely enabled structural solution via stabilisation of some of the inherent flexibility of the protein, and therefore allowing the alignment angles to be assigned more accurately in the cryoEM refinement, possibly via anchoring otherwise flexible loops into specific binding pockets. Alternatively, or in combination, with this stabilisation, the presence of additional asymmetric signal from the LPS may be sufficient to break the rotational averaging during alignment. It also remains possible that uncontrollable factors in the grid preparation serendipitously yielded a dataset capable of solving to high-resolution. This apparent stabilisation of FusA upon LPS binding is intriguing and gives hints about a possible role of LPS both in stabilisation of protein structure and modulation of activity via alteration of loop dynamics, as well as its well-known role in membrane integrity. Indeed, our observations are consistent with previous work showing that LPS can order the extracellular loops of OMPs (Nyenhuis et al., 2020, Balusek and Gumbart, 2016, Kucharska et al., 2016) and in stabilising or aligning OMPs into discrete OMP islands in the OM (Webby, 2022). Given this, we suggest that the addition of LPS may be a more general tool to use in attempting to solve the structure of relatively small OMPs by cryoEM, indeed the recently solved FhuA structure, another small OMP solved by cryoEM, also includes a stably bound and well-resolved LPS moiety (although this copurified rather than being added extraneously) (van den Berg et al., 2022).

It is well known that lipid molecules are important for the function of many membrane proteins, and are able to support specific structural motifs, enable functional transitions and to facilitate substrate recruitment and/or binding. Over the last decade, cryoEM has become an established method to elucidate protein-lipid interactions (Biou, 2023). However, these are typically identified either in nanodiscs, which tend to pose significant challenges for cryoEM structural determination and have a constrained possible lipid composition, or as strongly bound moieties that have been retained through a traditional detergent purification. Here we have shown that it is possible to reconstitute LPS of different length and complexity into detergent micelles, to distinguish them from micellar density and to show that they bind in defined sites, that in most (but not all cases) can be predicted using CG-MD. Therefore, we propose the addition of LPS into detergent-OMPs as a general route to facilitate their high-resolution structural solution by cryoEM. By using high-throughput cryoEM approaches coupled with lipid titrations of multiple lipid types, binding patterns and preferences could plausibly be determined.

The structures of several OMPs have been solved previously with LPS bound, including FhuA (Botte, 2022) (by both crystallography and cryoEM) and the OmpE36 trimer (Arunmanee, 2016) (crystallography, which showed two distinct binding sites). The structural impact of LPS binding, including loop stabilisation and the importance of LPS-tyrosine interactions, have been shown for OprH by NMR, but the lipid itself was not observed (Edrington et al., 2011). Further to the experimentally-resolved structures, multiple OMP-LPS interactions have been predicted by MD, including for BtuB, OmpA and OmpX (Shearer et al., 2019). The consequences of these OMP-LPS interactions on the OM’s permeability barrier remain unclear, although it has been proposed that binding of the antibiotic polymyxin B may significantly alter the conformational flexibility of LPS, and therefore presumably how it interacts with OMPs (Manioglu, 2022). Albeit at low resolution, the LPS-FusA binding sites revealed here via cryoEM and CG-MD reveal a range of LPS binding modes. Some of these modes have been observed before, including the importance of arginine, lysine and tyrosine for LPS-OMP binding (Shearer et al., 2019, Edrington et al., 2011) and proposed loop stabilisation upon LPS binding (Nyenhuis et al., 2020, Edrington et al., 2011),. However, others appear underappreciated, including the general importance of aromatic residues in stabilising binding and the importance of steric pockets. Further, the substantial agreement between the locations of LPS identified by cryoEM and MD is an encouraging validation of the ability of the Martini forcefield to simulate LPS-protein interactions accurately.

## Methods

### FusA purification

FusA was recombinantly produced in *E. coli* and purified from inclusion bodies as previously described (Grinter, 2016) with minor modifications. Briefly, N-terminally His_6_-tagged FusA from *P. atrosepticum* SCRI1043 (accession number: YP_048987) lacking its 20 amino acid signal sequence was overexpressed from *E. coli* BL21 (DE3) carrying the pET28a-based expression plasmid pFusA1043Δ20. Cells were grown in LB broth at 37 °C to an OD_600_ of 0.6, induced with 1 mM IPTG and grown overnight at 30 °C before harvesting by centrifugation. After cell lysis by sonication, the insoluble fraction was collected by centrifugation (22,000g, 30 min, 4 °C) and the inclusion bodies resuspended by homogenisation in 50 mM Tris, pH 7.5 containing 1.5 % LDAO. After incubation at room temperature for 30 min the inclusion bodies were pelleted by centrifugation and then sequentially washed by homogenisation and centrifugation in 50 mM Tris, pH 7.5 containing 1.5 % LDAO and 50 mM Tris, pH 7.5. The pelleted inclusion bodies were then solubilised by homogenisation in 10 mM Tris, 8 M urea, 1 mM EDTA, 1 mM DTT, pH 7.5 and incubated at 56 °C for 30 min. Insoluble material was then removed by centrifugation (8,000g, 10 min 4 °C). Refolding of FusA was achieved by drop wise addition, with stirring, to an equal volume of 20  mM Tris, 1  M NaCl, pH 7.9 containing 5 % LDAO. Refolded FusA was then dialysed at 4 °C overnight into 20  mM Tris, 0.5  M NaCl, pH 7.9 containing 0.1 % LDAO, applied to a 5 ml HisTrap FF column equilibrated in the same buffer and eluted with a 0–500 mM imidazole gradient. The eluted protein was applied to a HiLoad 26/600 Superdex 200 column equilibrated in 50  mM Tris, 200  mM NaCl, pH 7.9 containing 0.1 % LDAO and purified protein stored at −80 °C.

### Grid preparation

Purified FusA (50  mM Tris, 200  mM NaCl and 0.1 % (v/v) LDAO, pH 7.9) was diluted to ∼ 3 mg/ml. For the −FusA sample the protein was first buffer exchanged to 50  mM Tris, 200  mM NaCl and 0.05 % (w/v) DDM, pH 7.9) on a Superdex 200 10/300 column and concentrated to ∼ 3 mg/ml. For samples with LPS: Re-LPS (Sigma L9764) or Ra-LPS (Sigma L9641) was diluted in FusA buffer to 20x the final FusA concentration and then mixed 1:1 ratio with 2x concentrated FusA, and then left at room temperature for ∼ 30 min to allow for LPS equilibration. CryoEM grids were prepared using an optimised protocol (1.2/1.3 Quantifoil copper 300 mesh unless otherwise stated). Grids were glow-discharged for 20 s at 60 mA in a GlowQube Plus (Electron Microscopy Sciences) under amylamine vapour. 3 µl of sample was applied to each grid, incubated for 10 s, then blotted for 6 s with Whatman #1 filter paper at 4 °C and > 80 % humidity and plunge frozen into liquid ethane (Vitrobot Mark IV, Thermofisher). Collection 1 (K2 detector) was glow-discharged in air vacuum for 30 s at 60 mA, and the grids were blotted immediately after sample application.

### CryoEM data collection

Data was collected automatically on a 300 keV Titan Krios (ThermoFisher) TEM using EPU (Thermofisher), and using a range of different detectors (K2 + Gatan energy filter (10 eV slit width), Falcon4, Falcon4i, Falcon4i + Selectris energy filter (5 eV slit width)) as indicated for each sample. A 100 µm objective aperture was used in all cases. Full data collection parameters for each sample are shown in Supplementary Tables 1 and 2.

### CryoEM image processing and model building

Image processing was carried out in Relion 3.1 (Zivanov et al., 2020)/4.0 (Kimanius et al., 2021) unless otherwise stated. Dose-fractionated micrographs were motion-corrected by Relion and the CTF estimated by CtfFind4 (Rohou and Grigorieff, 2015). Collections 1–5 were initially picked using crYOLOs (Wagner, 2019) general model, and then subjected to either (A) 1–3 rounds of 2D classification and the best protein-like classes selected, or (B) 1–3 rounds of 2D classification followed by initial model generation and one round of 3D classification and particles from the best model selected. The ‘good’ particles selected from these approaches were used to train both crYOLO and topaz (Bepler, 2019) models, the datasets picked with a low-confidence threshold and the particle stacks combined and deduplicated. Particle counts for each of these stages are reported in Supplementary Table 1. As described in the main text, the reconstructions for model 1–6 showed varying extents of rotational averaging, and thus the reported resolutions are estimates only. All map sharpening and local resolution estimates used Relion.

For collection 1: 361,633 particles from trained picking models were passed through two rounds of 2D classification, leaving 68,263 good protein particles, which were unbinned, in a 280 pixel box and used to generate an initial model. Multiple rounds of iterative 3D classification and 3D refinement resulted in a final particle stack of 10 623, and a model of ∼ 13 Å.

Collection 2: 417,907 particles from trained picking models were passed through three rounds of 2D classification leaving 103,092 particles which were extracted and unbinned into a 288 pixel box. Following initial model generation, a single round of 3D classification yielded 31,357 particles which refined to a model of ∼ 8.6 Å. Bayesian polishing and CTF refinement made no improvements to the map.

Collection 3: 896,632 particles from trained picking models were 2D classified once using both VDAM and EM algorithms and the good classes from each recombined and further 2D classified to give 152,767 particles, which were extracted into a 288 pixel box. 3D refinement gave the best model of ∼ 9.7 Å. Further splitting of the particle stack by 3D classification did not improve the model. 3D refinement of topaz denoised data yielded a map of similar quality.

Collection 4: 377,986 particles from trained picking models were 2D classified once using VDAM algorithm to remove junk, and all okay particles then 2D classified with multiple parameters (VDAM or EM, T value (2–8), with/without ignoring CTF to first peak) in parallel. The best classes were recombined to 94,277 particles which were extracted into a 240 pixel box. Following 2 rounds of 3D classification 46,189 particles were 3D refined (to ∼ 9 Å), and an additional round of 3D classification yielded 31,872 particles. A single round of polishing and masked refinement gave the best model of ∼ 8.6 Å.

Collection 5: 214,571 particles from trained picking models were 2D classified twice, giving 34,105 particles. Particles were extracted unbinned in a 288 pixel box and 3D classified once. The resultant 32,329 particles refined to final model of ∼ 18 Å resolution.

Collection 6: 711,757 particles were picked by crYOLO’s general model and extracted with 2x binning for two rounds of 2D classification. Good classes were grouped into side, top and tilt views and each group used to train a new crYOLO and topaz model, to repick the data at a low threshold (finding 1, 237,542 unique particles). Following one round of 2D classification to remove junk, all good particles were then subjected to two rounds of 2D classification with multiple parameters (T value 2 or 8), with/without ignoring CTF to first peak) in parallel. The best classes were recombined and deduplicated to 275,399 particles, 3D classified twice, leaving 124,599 good particles. (An additional lower resolution class was identified that appeared to be missing the plug domain entirely). These were iteratively refined and classified to leave 85,859 particles that yield a ∼ 10 Å reconstruction, which were then filtered to 74,343 particles by excluding all the particles whose angular assignment had rotated by more than 10° in any Euler angle during the final iteration of 3D refinement. An additional round of 3D refinement on these particles gave a final model of ∼ 8.3 Å resolution. Polishing and CTF refinement yielded no improvement to the model.

Collection 7: 545,970 particles were picked by crYOLO’s general model, extracted with 2x binning and 2D classified twice. Good class averages were grouped as top, tilt or side views, each subject to additional round of 2D classification and then the good classes from each group used to train a new crYOLO and topaz model. These were each subjected to two rounds of 2D classification with multiple parameters (T value (2 or 8), with/without ignoring CTF to first peak, EM or VDAM algorithm) in parallel. The protein-like classes that resulted were recombined and deduplicated to yield 261,382 particles. Following two rounds of 2D classification a final high-resolution 37,826 particle stack was passed into 3D refinement, giving a model of ∼ 8.6 Å. Following one round of polishing and CTF refinement, a final reconstruction at ∼ 7.2 Å was obtained. The map was sharpened with b-factor of −187.6 Å^2^. Neither additional 3D classification or cryoSPARCs Non-uniform (Punjani et al., 2020) refinement improved the model.

Collection 8: 754,788 particles were picked by crYOLO’s general model, extracted with 2x binning and passed through 2D classification, giving 183,330 protein-like particles. These were used to train a crYOLO model which gave 360,633 particles, which were subject to iterative, divisive 2D classification (where groups of similar views were 2D classified on separately through multiple rounds). Top, side and tilt views were then re-grouped and used individually to train crYOLO and topaz models, picked with a low threshold and the deduplicated particle stack from each of the view groups subject to iterative, divisive 2D classification. This eventually yielded 38,494 excellent particles for initial model generation, and were then passed directly into 3D refinement, yielding a model of ∼ 6 Å. This model was used for multiple rounds of 3D classification with all the crYOLO/topaz picked particles, giving a final particle number of 84, 061. Following box expansion to 400 pixels, these were then subjected to 5x rounds of cryoSPARC Non-Uniform (Punjani et al., 2020) refinement and Relion polish and CTF refinement. Postprocessing and sharpening in relion gave a final model of 2.8 Å resolution. Applying a tighter mask during postprocessing resolved higher resolution protein features in the plug and barrel domains. This map was used to create Fig. 3 and is deposited as a secondary map.

3D classification/refinement of the optimised particle stacks from collections 1–6 against the high resolution Fusa:Ra-LPS model from collection 8 did not yield improved reconstructions.

### Model building

The FusA crystal structure (4ZGV) (Grinter, 2016) was used as a starting point and following optimisation in ISOLDE (Croll, 2018) the model was passed through real-space refinement in PHENIX (Afonine, 2018) (v1.20) with secondary structure restraints and manually optimised in COOT (Emsley et al., 2010). Geometry was assessed using molprobity (MolProbity). For LPS modelling (and display), non-protein density was segmented from the map, (and for display smoothed using vop-gaussian in chimera (Goddard, 2018), then manually inspected. Additional density seen around the upper parts of FusA’s extracellular loops likely reflect the lower resolution and higher mobility of these regions, rather than true LPS binding, with the protein model fit being substantially more uncertain in these regions compared to the transmembrane and membrane-adjacent regions of the barrel.

### Molecular dynamics

Coarse-grained models of FusA were generated from the crystal structure (PDB: 4ZGV (Grinter, 2016)) using the martinize script and an elastic spring network of 1000 KJ/mol/nm^2^ (upper distance cut-off of 0.7 nm). CG-MD simulations were performed with GROMACS (Van Der Spoel, 2005) (v5.0.7) using the Martini (v2.2) forcefield (Marrink et al., 2007, de Jong, 2013). Bilayers were built using the insane script (Wassenaar et al., 2015) to randomly place phospholipids. Initial simulations with randomly distributed 25 % LPS were found not to converge over 20 µs. Thus, 10 LPS molecules were placed around FusA using a custom script that removed 3 phospholipids from the membrane at a specified location and replaced them with a single LPS moiety. The system was neutralised with 0.1 M NaCl and 0.025 M CaCl_2_, energy minimised (steepest descent algorithm) and equilibrated with the protein backbone position-restrained for 3 ns. The final snapshot of the equilibrated system was used to generate seeds for production simulations. Productions simulations were run for 10 / 20 us (Table S3) with a 20 fs time-step for Re-LPS containing systems and with a 10 fs time-step for Ra-LPS containing systems, and frames were generated at 200 ps intervals. The barostat and thermostat were Parinello-Rahman (Parrinello and Rahman, 1981) (1 bar) and V-rescale (Bussi et al., 2007) respectively. A compressibility of 3 × 10^-4^ bar^−1^ was used. The LINCS algorithm constrained bond lengths (Hess et al., 1997).

Lipid-protein contact analysis used a 0.55 nm distance cutoff to define contacts, performed on merged data from all replicas using gmx mindist. All lipid-protein contacts were normalised to lipid number of each lipid type and simulation time. For lipid density analysis the trajectories of all simulation replicas were concatenated and the protein orientation centered and fixed (gmx trjconv), gmx densmap was used to calculate densities. MSD and RDF analysis used gmx msd and gmx rdf respectively. Residence time was calculated using the pyLipID (Song, 2022) module with short and long distance cutoffs of 0.475 and 0.8 nm. LPS binding poses were determined using pyLipID (different poses that were at the same interaction site were combined for calculation of the site occupancy). Lipid binding sites in the contact analysis were determined by taking peaks that were at least 2σ above the non-zero background, and clustering contact sites if they were in adjacent β-strands. The length of the Ra-LPS polysaccharide was determined with gmx mindist between beads S01 and S39. Protein-lipid contacts shown in the results represent data merged from all simulation repeats of that system. Additionally, for each system, these contacts were compared between individual simulations to ensure some consistency in binding sites. Simulations were visualised using VMD (Humphrey et al., 1996) and Blender (https://www.blender.org) with a set of custom scripts (github.com/JonMarks29/Biomolecular-Blender).

## CRediT authorship contribution statement

**Jonathan M. Machin:** Conceptualization, Data curation, Formal analysis, Investigation, Methodology, Validation, Visualization, Writing – original draft, Writing – review & editing. **Khedidja Mosbahi:** Resources. **Dheeraj Prakaash:** Methodology, Investigation, Formal analysis, Data curation. **Sheena E. Radford:** Writing – review & editing, Writing – original draft, Supervision, Project administration, Investigation, Funding acquisition, Conceptualization. **Daniel Walker:** Writing – review & editing, Resources, Conceptualization. **Antreas C. Kalli:** Writing – review & editing, Writing – original draft, Supervision, Project administration, Methodology, Investigation, Formal analysis, Conceptualization. **Neil A. Ranson:** Conceptualization, Formal analysis, Funding acquisition, Methodology, Project administration, Supervision, Writing – original draft, Writing – review & editing.

## Declaration of competing interest

The authors declare the following financial interests/personal relationships which may be considered as potential competing interests: Neil Ranson reports financial support was provided by Wellcome Trust. Neil Ranson reports financial support was provided by UK Research and Innovation Medical Research Council. Jonathan Machin reports financial support was provided by Wellcome Trust. Sheena Radford reports was provided by The Royal Society. If there are other authors, they declare that they have no known competing financial interests or personal relationships that could have appeared to influence the work reported in this paper.

## Data Availability

Source data files containing thinned MD trajectories and scripts used to generate initial MD files are freely available at the University of Leeds Data Repository (https://doi.org/10.5518/1668).
