# Supplementary material for: The structure of the bacterial outer membrane transporter FusA enabled by addition of the native lipid lipopolysaccharide
Source: J Struct Biol X. 2025 Nov 18;12:100141. doi: 10.1016/j.yjsbx.2025.100141 (PMC12702245; doi:10.1016/j.yjsbx.2025.100141)
Supplement: Supplementary Data 1 [file mmc1.docx]

**Supplementary Figures**


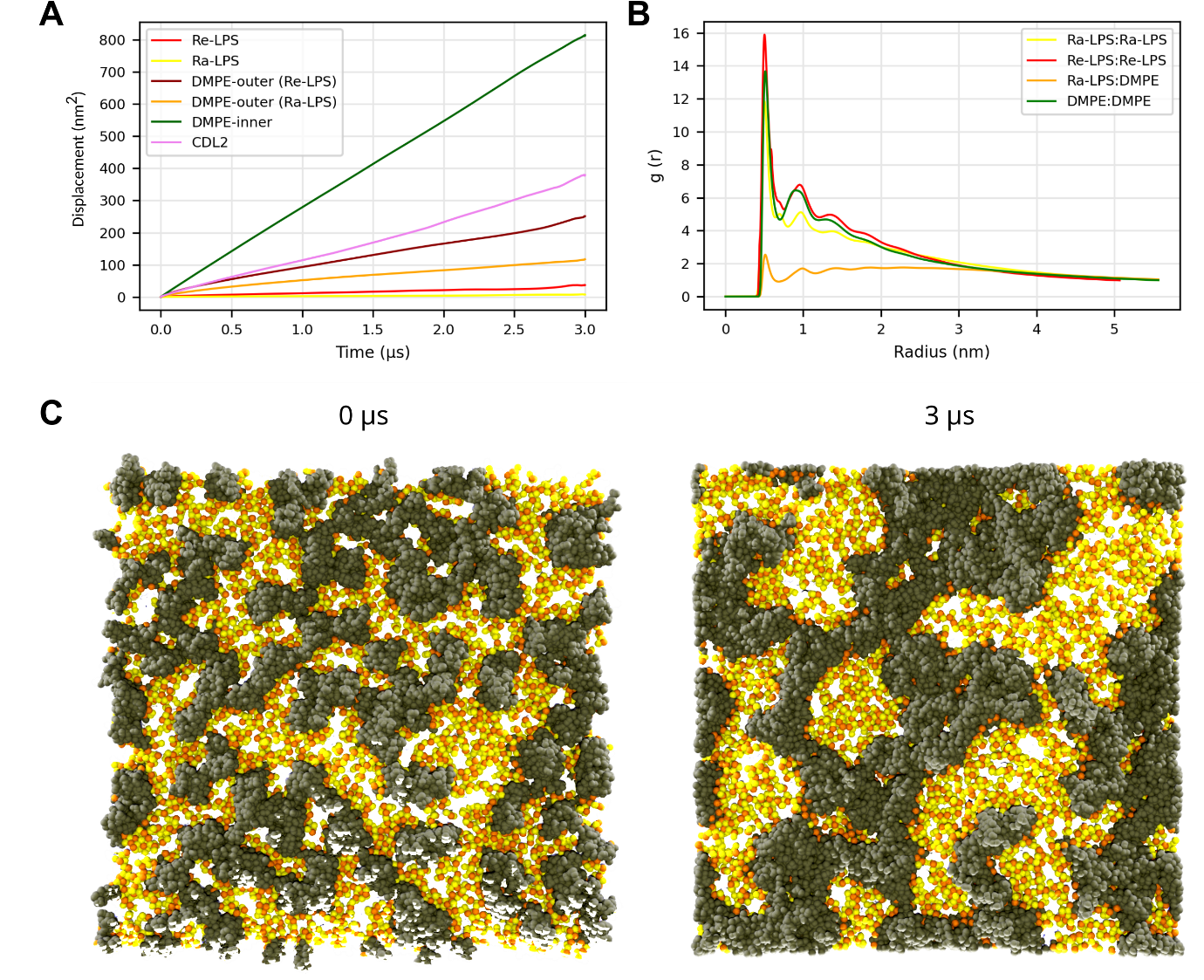


**Supplementary Figure 1: Coarse-grained LPS model properties in lipid only simulations.** (**a**) Mean squared displacement of LPS and phospholipid species over 3 μs simulations. Re-LPS and Ra-LPS were simulated in separate systems. Inner leaflet DMPE and CDL2 are shown for the Ra-LPS simulation only, but they were similar with both compositions. (**b**) Radial distribution functions of LPS-LPS, LPS-DMPE and DMPE-DMPE calculated over the whole simulation trajectory LPS-DMPE and DMPE-DMPE are shown for Ra-LPS simulation only, but they were similar with both compositions. (**c**) Initial and end-point frames for one replica of the 25% Ra-LPS simulation (dark-grey: LPS core, yellow/green: DMPE head-group, orange: phosphates) showing clear LPS clustering.


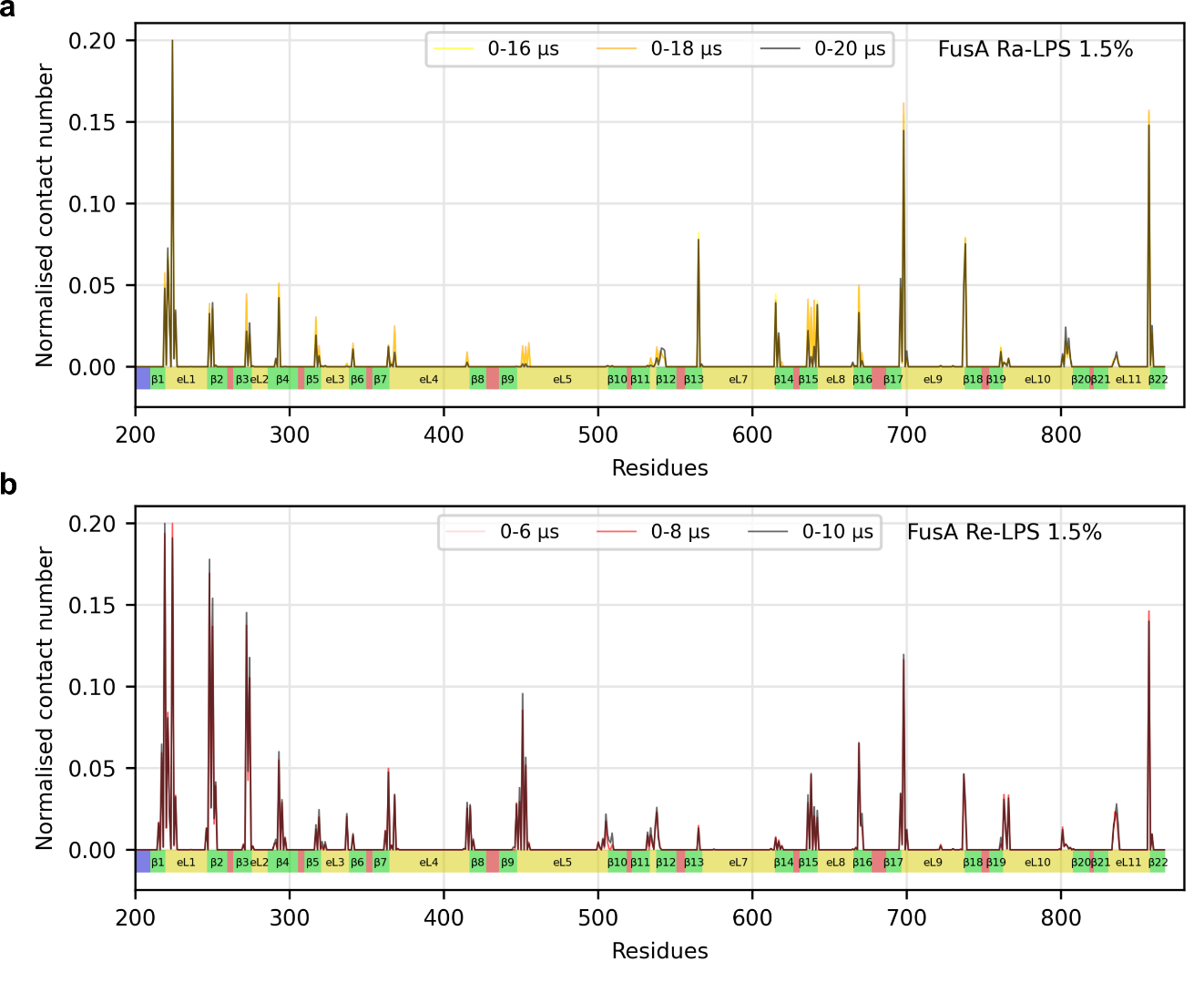


**Supplementary Figure 2: Simulation lipid-protein contact convergence.** The normalised lipid-protein interactions (number of interactions between each type of lipid and each protein residue normalised by lipid type number and number of frames) are approximately the same comparing with and without the final 4 µs or 2 µs of simulations with outer leaflets containing (**a**) 1.5% Re-LPS, and (**b**) 1.5% Ra-LPS, indicating contact convergence.


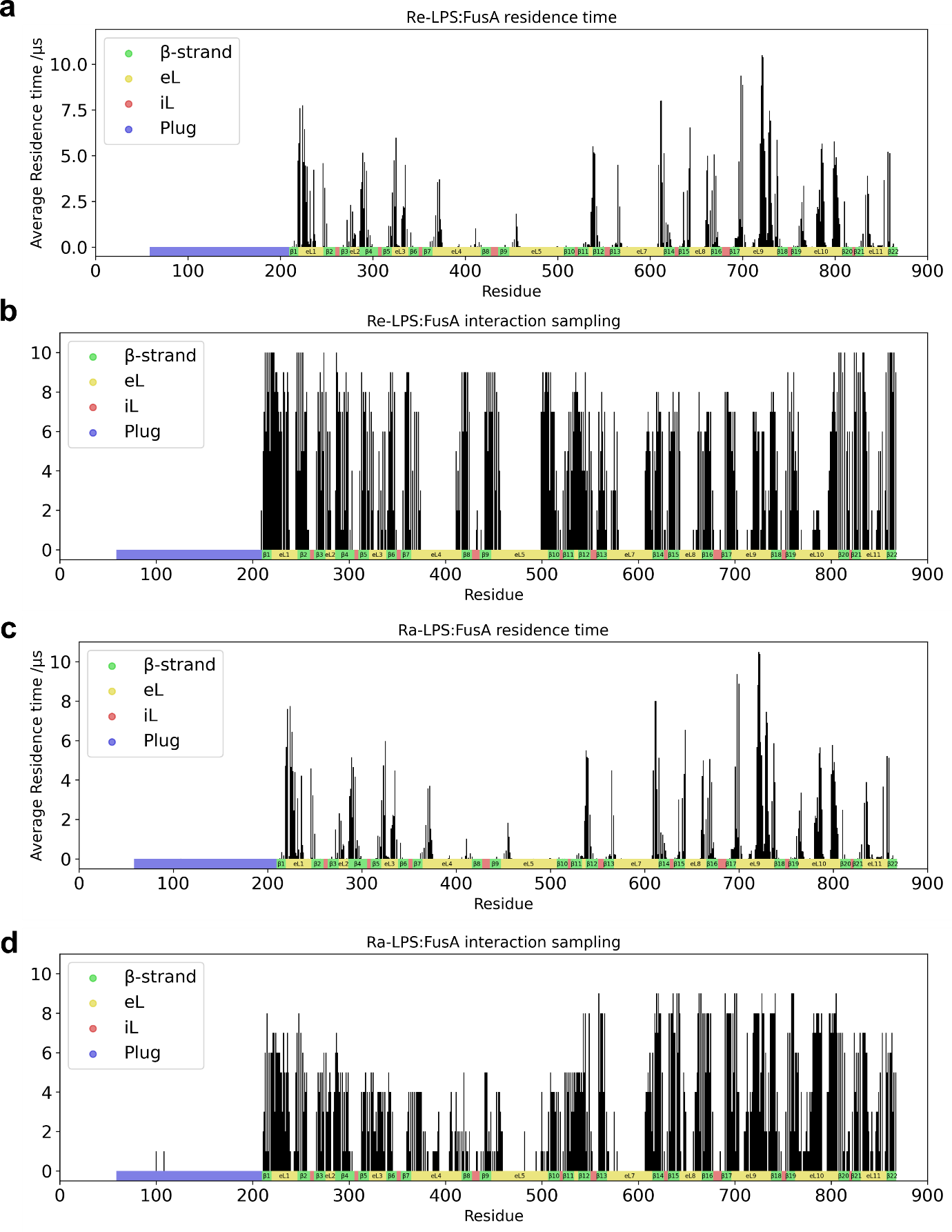


**Supplementary Figure 3: Sparse-LPS simulation lipid-protein sampling and binding residences times.** (**a**) Average residence time of Re-LPS per residue of FusA over all replicates of the ~1.5% Re-LPS simulation. (**b**) Re-LPS:FusA sampling shown by the number of replicates where an LPS-FusA contact has been made (for any length of time). All expected protein regions were sampled across multiple replicates. (**c**) and (**d**) as (a) and (b) for Ra-LPS.


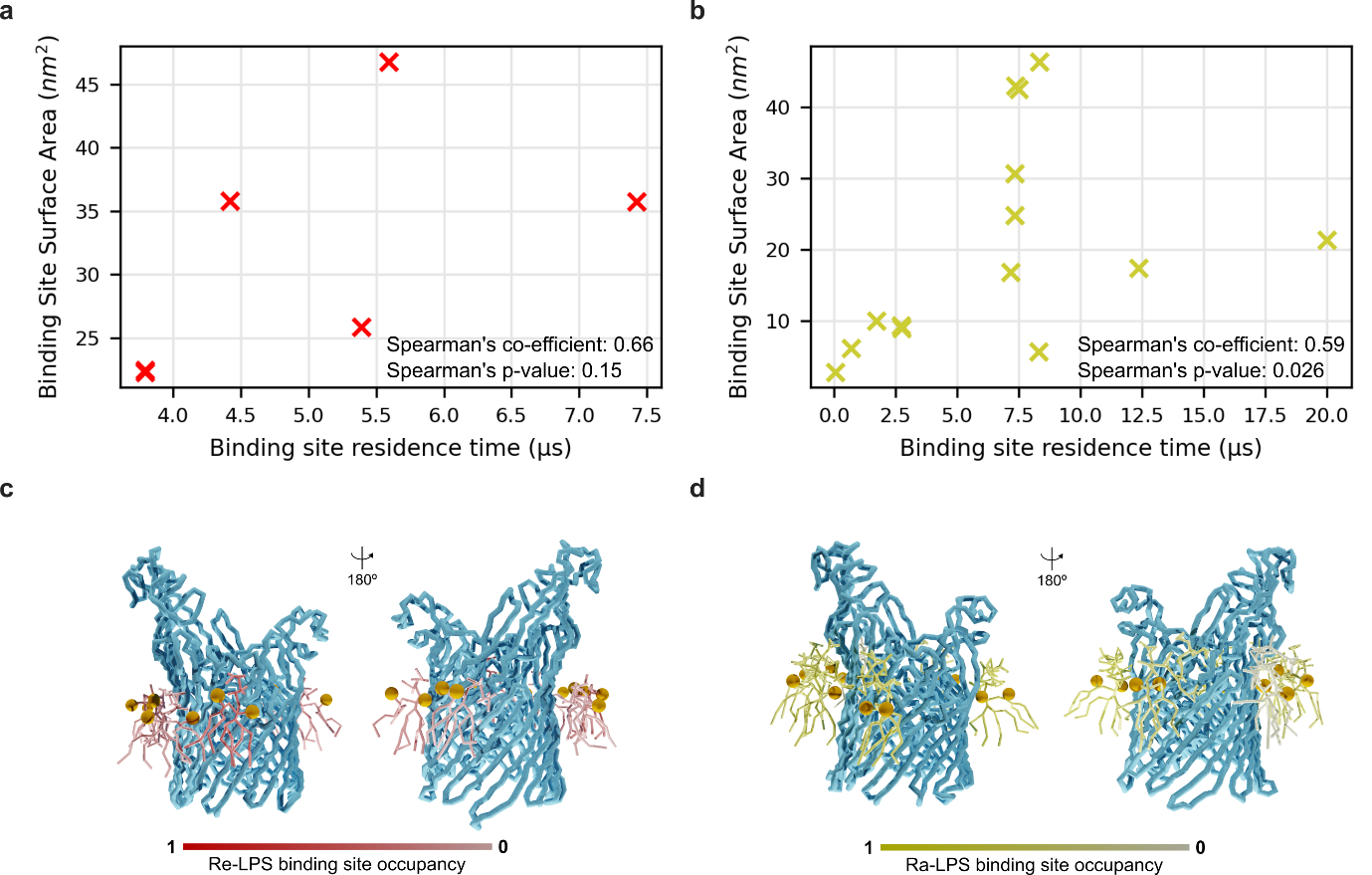


**Supplementary Figure 4: pyLipID analysis of Re-LPS and Ra-LPS binding poses.** (**a**) Re-LPS and (**b**) Ra-LPS binding site residence time by binding site surface area for all binding poses, including those at the same sites. Summary of non-overlapping binding poses of (**c**) Re-LPS (red) and (**d**) Ra-LPS (green), coloured by their relative, normalised binding site occupancy over the simulation time.


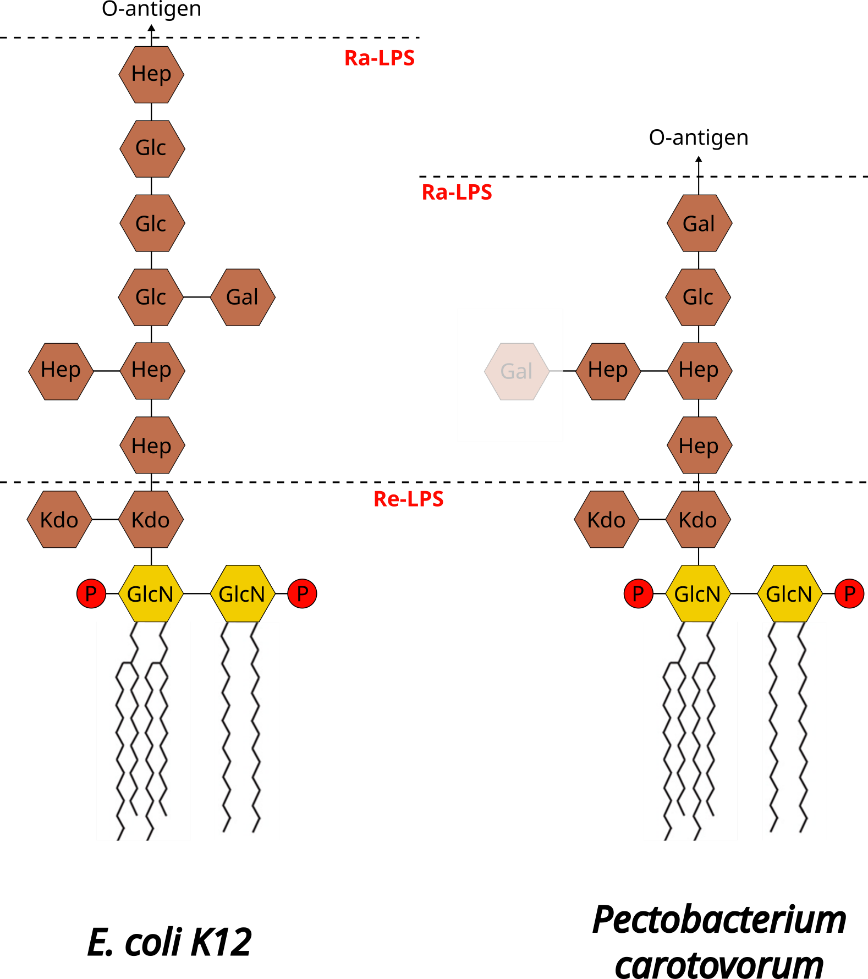


**Supplementary Figure 5:** Comparison between the sugar composition and organisation of Ra-LPS and Re-LPS used in this study (from K12 *E. coli*) and that of *Pectobacterium carotovorum*. The half-coloured Gal in *Pectobacterium* was identified substoichiometrically via mass spectrometry^37^. (GlcN: D-glucosamine, Kdo: 2-keto-3-deoxyoctulosonate, Hep: heptose, Glc: glucose, Gal: galactose).


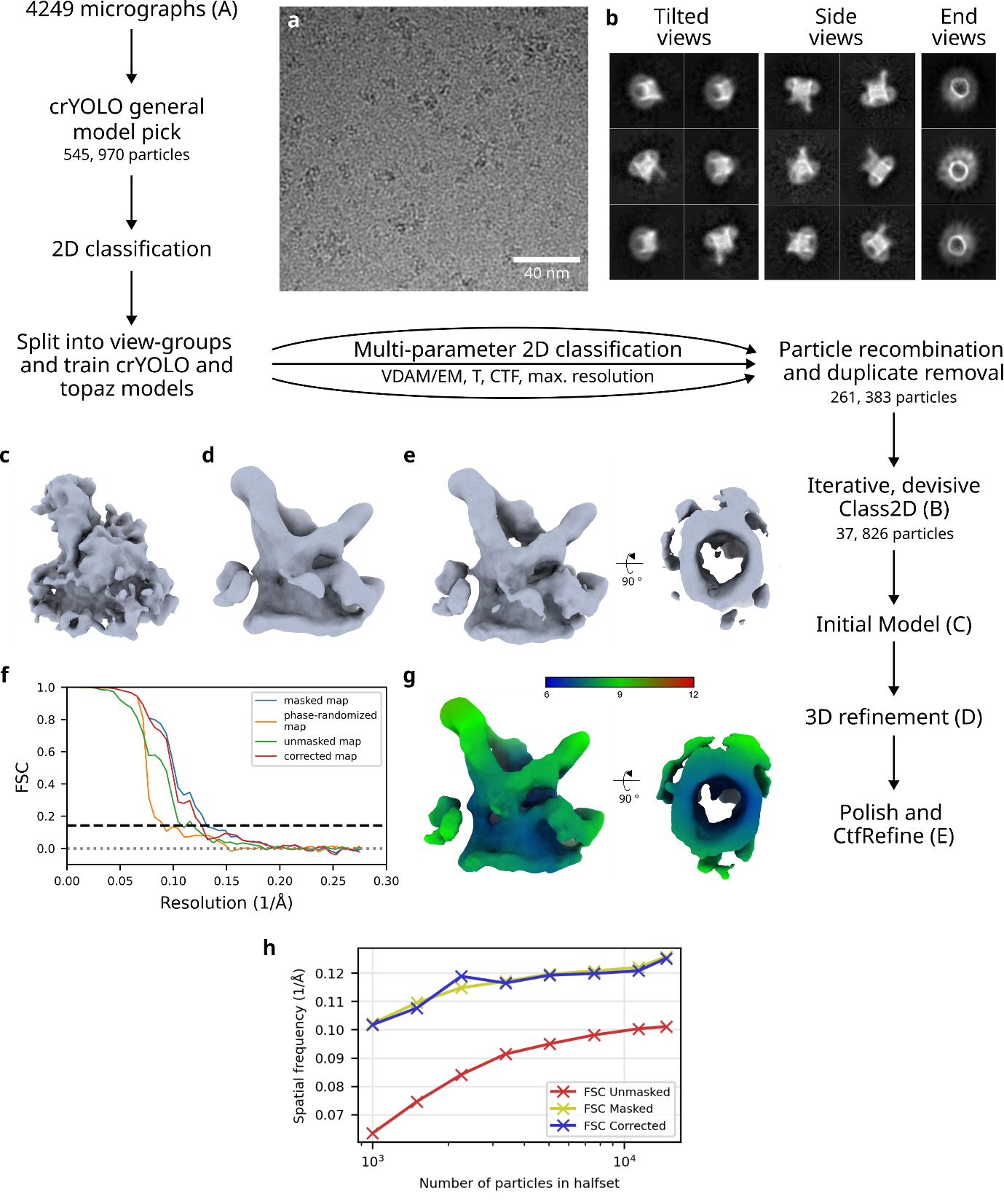


**Supplementary Figure 6: CryoEM processing workflow for FusA:Re-LPS.** Micrographs (**a**) were picked using crYOLO’s general model, and the resulting 2D classes were split into views and used to train new models. Following parallel multi-parameter 2D classification, good particles were combined and additional 2D classification performed, resulting in excellent classes (**b**). Initial model generation (**c**) yielded a broadly correct reconstruction (**c**), improved via 3D refinement (**d**), as well as particle polishing and CTF refinement (**e**). Together this resulted in a model ~8Å resolution, shown in the (**f**) FSC curves and (**g**) local resolution maps. (**h**) Reslog plot showing effect of particle count on resolution (FSC cutoff: 0.143).


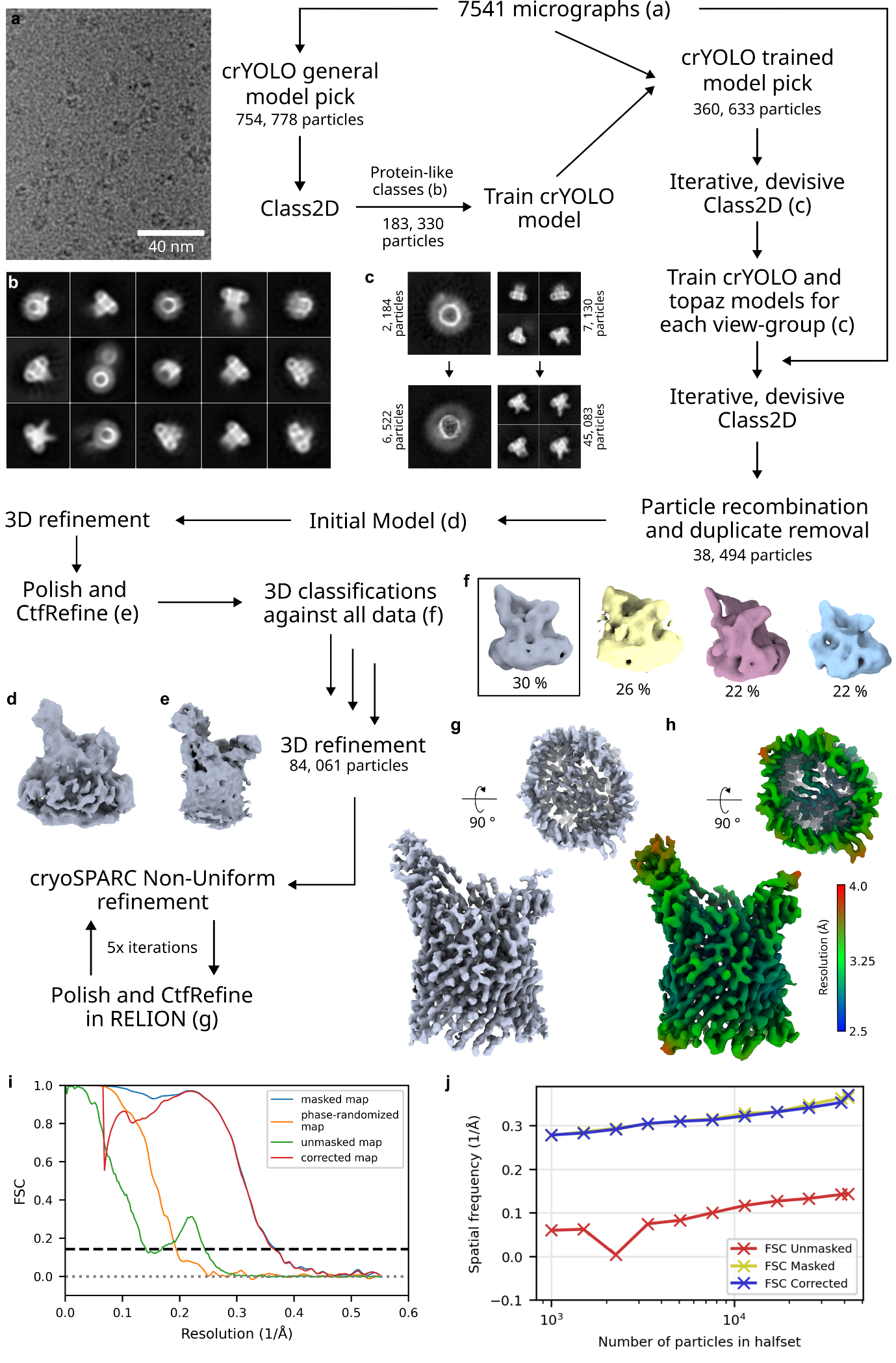


**Supplementary Figure 7: CryoEM processing workflow for FusA:Ra-LPS.** Micrographs (**a**) were picked using crYOLO’s general model, and the resulting protein-like 2D classes (**b**) were used to train a crYOLO model, particles from which were 2D classified and split into view groups to train additional crYOLO/topaz models, yielding higher particle counts for each view (**c**). Initial model generation (**d**) and 3D refinements (**e**) yielded broadly correct but low-resolution models. These models were used to run multiple rounds of 3D classification against all the data (**f**). The optimised particle stack was subjected to five cycles of cryoSPARCs Non-Uniform refinement and polishing/CTF refinement in relion, yielding the final maps (**g**), with local resolution estimates (**h**). (**I**) The FSC curves of the model. (**j**) Reslog plot showing effect of particle count on resolution (FSC cutoff: 0.143).


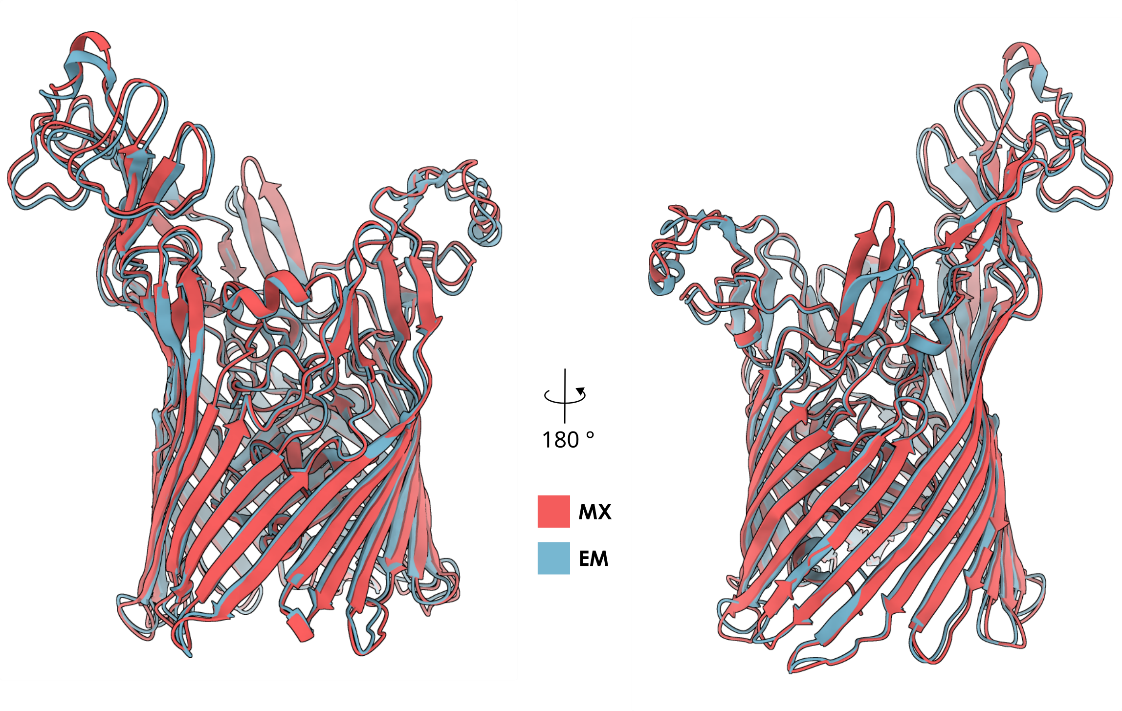


**Supplementary Figure 8: FusA crystal structure compared to FusA cryoEM structure.** Models are very similar (average RMSD: 1.2 Å), with some flexibility in the extracellular loops. (Cystal structure PDB: 4ZGV^31^).


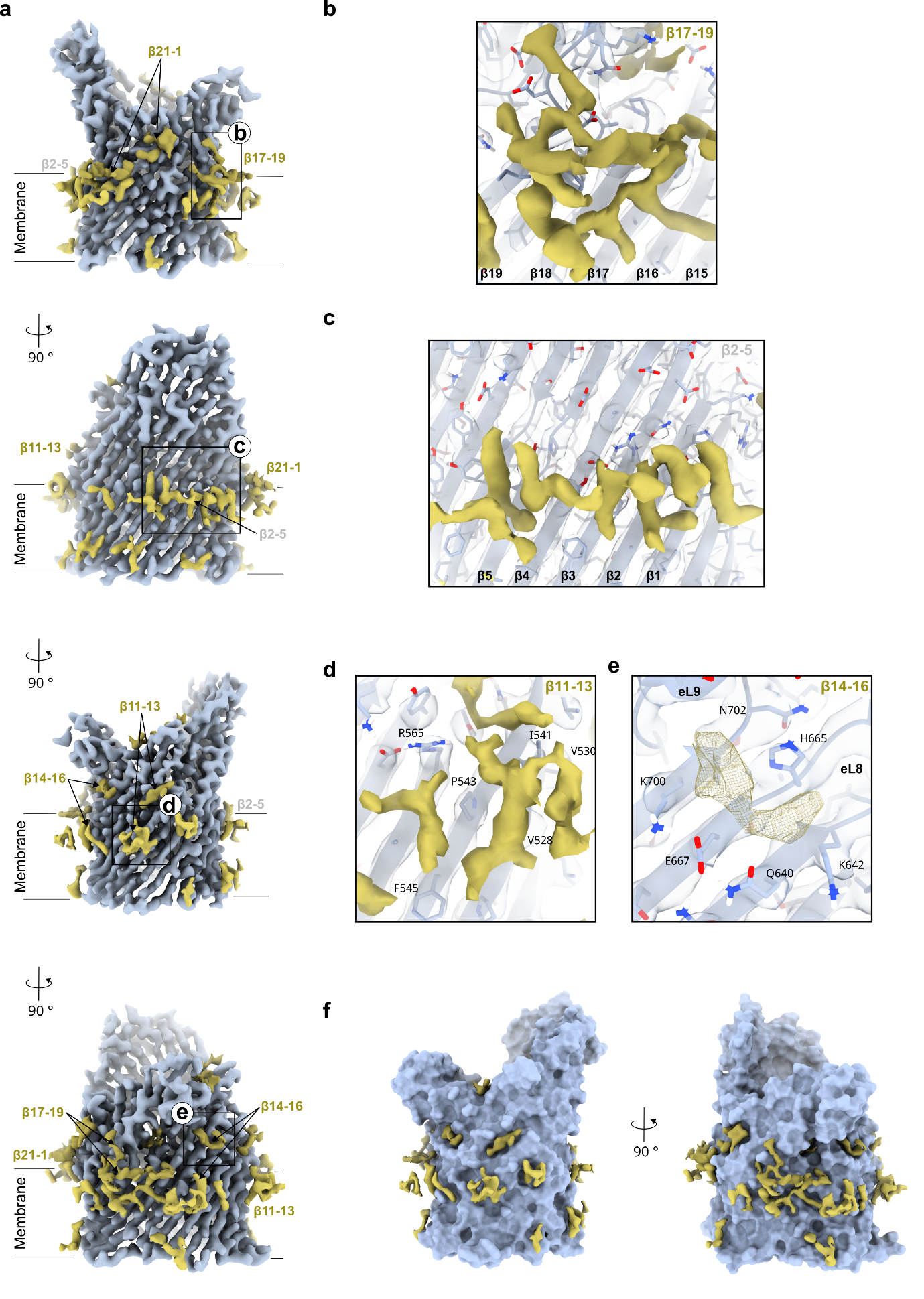


**Supplementary Figure 9: Additional features of FusA:Ra-LPS reconstruction.** (**a**) Overview of model showing protein density (blue) and non-protein density (green) turned at 90 ° increments. Assigned LPS binding sites are labelled as in main text. (**b**)-(**e**) Additional detail of the non-protein density at different sites as indicated in (**a**) (see also upper right of each panel). (**b**) and (**e**) are extra-micellar densities for binding sites at β17-19 and β14-16 respectively. β17-19 is less well defined and appears as a network of density. (**c**) and (**d**) are the acyl-chains at sites β2-5 and β11-13 respectively. Note that β2-5 had no additional extra-micellar density. (**f**) Representing the protein in a space-filling view reveals that many of the resolved assigned LPS densities fit within grooves or pockets on the protein surface.


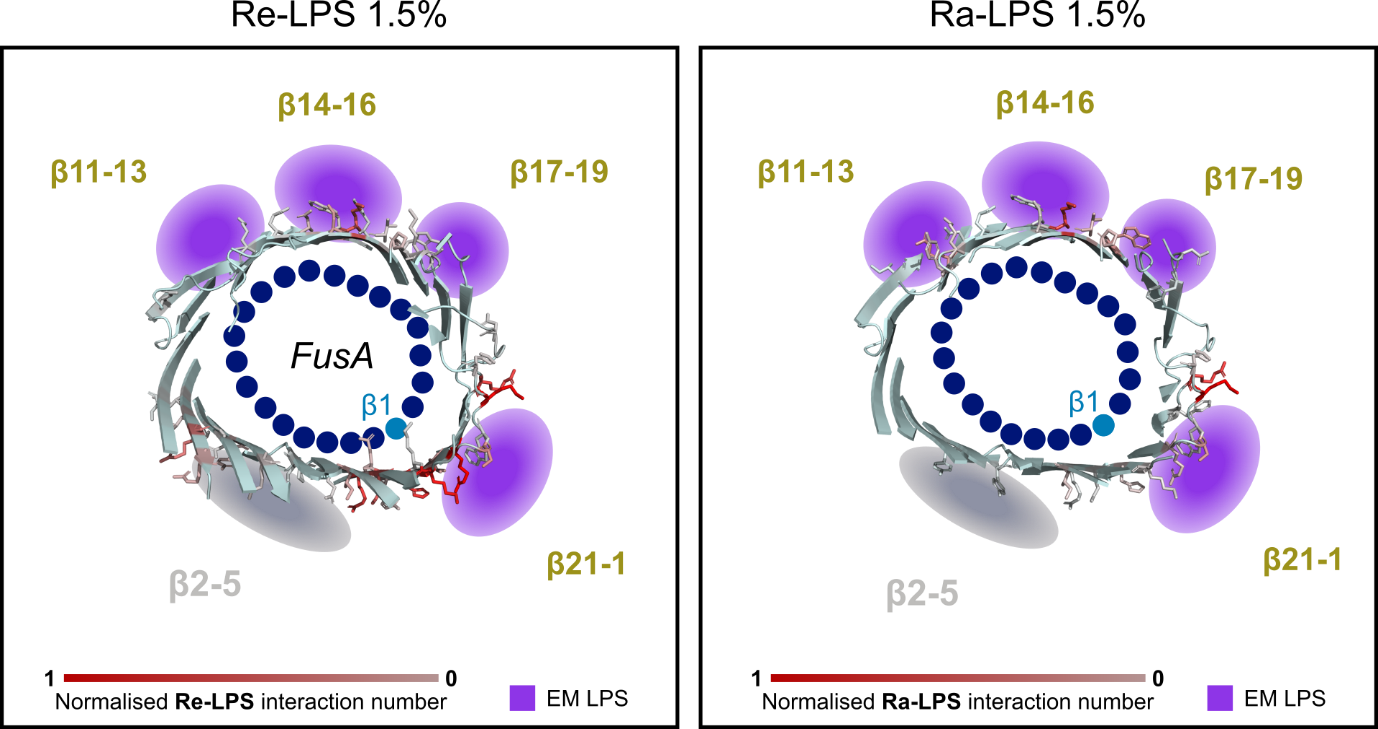


**Figure S10: Comparison of Ra-LPS binding to FusA by CG-MD and cryoEM.** Interacting residues for Re-LPS (left) and Ra-LPS (right) simulations coloured by the normalised contact count (from LPS Lipid A phosphate beads to the protein) (both lipids shown as pink-red for clarity), mapped onto the fitted FusA model. FusA transmembrane strands are shown in blue and the location of β-strands indicated by blue dots, progressing in a clockwise direction from β1 to β22. The approximate locations of the LPS binding sites inferred from the cryoEM are indicated by purple spots and labelled as in the main-text. The grey spot at β2-5 is where acyl-chains were observed binding without additional non-micellar density. Generally good agreement between the EM patches and the interacting residues, as well as the higher contact counts, is observed. Similar to the pyLipID pose analysis (**Figure 4**), a better agreement to the cryoEM is observed with the Re-LPS.

**Supplementary Tables**

Supplementary Table 1 | CryoEM data collection statistics for low-resolution reconstructions

|  | Collection 1  K2 detector  Gatan filter (20 eV) | Collection 2  K2 detector  Gatan filter (20 eV) | | Collection 3  F4 detector | Collection 4  F4 detector  DDM-FusA | Collection 5  F4 detector  Low defocus | Collection 6  F4i detector  Selectris (5 eV) | Collection 7  F4i detector  Selectris (5 eV)  10x Re-LPS |
| --- | --- | --- | --- | --- | --- | --- | --- | --- |
| **Data collection and processing** |  | |  |  |  |  |  |  |
| Magnification | 130k | | 130k | 130k | 96k | 96k | 130k | 130k |
| Voltage (kV) | 300 | | 300 | 300 | 300 | 300 | 300 | 300 |
| Electron exposure (e–/Å^2^) | 62.0 | | 66.12 | 40.0 | 40.0 | 40.0 | 45.11 | 47.1 |
| Defocus range (μm) | -1.0 to -2.5 | | -1.0 to -2.5 | -1.0 to -3.0 | -1.0 to -3.0 | -0.25 to -2.0 | -0.9 to -3.0 | -0.9 to -3.0 |
| Pixel size (Å) | 1.07 | | 1.07 | 0.91 | 0.83 | 0.83 | 0.91 | 0.91 |
| Symmetry imposed  Micrograph number | C1  892 | | C1  3235 | C1  3584 | C1  1336 | C1  1846 | C1  7511 | C1  4249 |
| Initial particle images (no.)  Picking model training particle images (no.)  Model picked particles (no.) | 72 358  37 132  361 633 | | 286 338  5 804  417 907 | 279 457  54 706  896 632 | 377 986  17 369  377 986 | 236 914  28 157  214 571 | 711 757  -  - | 545 970  -  - |
| Final particle images (no.) | 10 623 | | 31 357 | 76 383 | 31 872 | 32 329 | 74 343 | 37 826 |
| Map resolution (Å)  0.143 FSC threshold | 13 | | 8.6 | 9.7 | 8.6 | 18 | 8.3 | 7.2 |
|  |  | |  |  |  |  |  |  |

Supplementary Table 2 | CryoEM data collection, refinement, validation and model building statistics for FusA:Ra-LPS (collection 8)

|  | FusA:Ra-LPS  (EMDB- 53974)  (PDB_00009RHR) |
| --- | --- |
| **Data collection and processing** |  |
| Magnification | 130k |
| Voltage (kV) | 300 |
| Electron exposure (e–/Å^2^) | 47.1 |
| Defocus range (μm) | -0.9 to -3.0 |
| Pixel size (Å) | 0.91 |
| Symmetry imposed | C1 |
| Initial particle images (no.) | 754 778 |
| Final particle images (no.) | 84 061 |
| Map resolution (Å)  0.143 FSC threshold | 2.4 |
| Map resolution range (Å) | 2.2-4.2 |
|  |  |
| **Refinement** |  |
| Initial model used (PDB code) | 4ZGV |
| Model resolution (Å) | 3.2 |
| Map sharpening *B* factor (Å^2^) | Local sharpening |
| Model composition  Non-hydrogen atoms  Protein residues  Ligands | 12476  809  4 |
| *B* factors (Å^2^)  Protein | 0 |
| R.M.S. deviations  Bond lengths (Å)  Bond angles (°) | 0.003  0.595 |
| Validation  MolProbity score  Clashscore  Favored rotamers (%)  Poor rotamers (%) | 1.02  0.88  96.87  0.14 |
| Ramachandran plot  Favored (%)  Allowed (%)  Disallowed (%) | 96.28  4.72  0 |

**Supplementary Table 3: Summary of MD simulations**

| **Protein** | **Membrane composition** | **Box size /nm** | **Time /µs** | **Replicas** |
| --- | --- | --- | --- | --- |
| - | Outer: 25:75 **Re-LPS**:DLPE  Inner: 80:15:5 DLPE:DLPG:CDL2 | 40x40x11 | 3 | 3 |
| - | Outer: 25:75 **Ra-LPS**:DLPE  Inner: 80:15:5 DLPE:DLPG:CDL2 | 40x40x12 | 3 | 3 |
| FusA | Outer: 1.5:98.5 **Re-LPS**:DLPE  Inner: 80:15:5 DLPE:DLPG:CDL2 | 20x20x16 | 10 | 10 |
| FusA | Outer: 1.5:98.5 **Ra-LPS**:DLPE  Inner: 80:15:5 DLPE:DLPG:CDL2 | 20x20x16 | 20 | 10 |
